# Acquired Enamel Pellicle Engineered Peptides: Effects on Hydroxyapatite Crystal Growth

**DOI:** 10.1038/s41598-018-21854-4

**Published:** 2018-02-28

**Authors:** Maria Teresa Valente, Eduardo Buozi Moffa, Karla Tonelli Bicalho Crosara, Yizhi Xiao, Thais Marchini de Oliveira, Maria Aparecida de Andrade Moreira Machado, Walter Luiz Siqueira

**Affiliations:** 1 School of Dentistry and Department of Biochemistry, Schulich School of Medicine & Dentistry, The University of Western Ontario, London, ON Canada; 2 Department of Pediatric Dentistry, Orthodontics and Public Health, Bauru Dental School, University of São Paulo, Bauru, Brazil

## Abstract

The aim of this study was to test the hypothesis that duplication/hybridization of functional domains of naturally occurring pellicle peptides amplified the inhibitory effect of hydroxyapatite crystal growth, which is related to enamel remineralization and dental calculus formation. Histatin 3, statherin, their functional domains (RR14 and DR9), and engineered peptides (DR9-DR9 and DR9-RR14) were tested at seven different concentrations to evaluate the effect on hydroxyapatite crystal growth inhibition. A microplate colorimetric assay was used to quantify hydroxyapatite crystal growth. The half-maximal inhibitory concentration (IC_50_) was determined for each group. ANOVA and Student-Newman-Keuls pairwise comparisons were used to compare the groups. DR9-DR9 increased the inhibitory effect of hydroxyapatite crystal growth compared to single DR9 (*p* < 0.05), indicating that functional domain multiplication represented a strong protein evolution pathway. Interestingly, the hybrid peptide DR9-RR14 had an intermediate inhibitory effect compared to DR9 and DR9-DR9. This study used an engineered peptide approach to investigate a potential evolution protein pathway related to duplication/hybridization of acquired enamel pellicle’s natural peptide constituents, contributing to the development of synthetic peptides for therapeutic use against dental caries and periodontal disease.

## Introduction

Saliva is mainly formed by the secretion of salivary glands. The major functions of saliva are lubrication and protection of oral tissues, buffering, oral clearance, and immune defense. Whole saliva represents a combination of components derived from salivary glands, gingival crevicular fluid, food debris, and oral microorganisms and their substances^1,2^. Saliva is a complex mixture of organic components, which are predominantly salivary proteins and peptides, and inorganic components, which are predominantly calcium and phosphate ions related to the remineralization process of dental enamel, preventing dental caries and/or dental erosion^2–5^. The concentration of inorganic ions in human saliva indicates a state of supersaturation with respect to hydroxyapatite (HA) crystal, the major component of tooth enamel^6,7^. This supersaturated environment in saliva is the driving force for secondary mineralization (remineralization) of the enamel surface. However, this process is strictly regulated by salivary proteins, particularly those that form the acquired enamel pellicle (AEP), a thin protein layer formed by the selective adsorption of proteins and peptides on the enamel surface that protects the tooth against enamel demineralization^8,9^. This proteinaceous film also prevents the deposition of calcium and phosphate on the surface of the enamel and promotes the inhibition of dental calculus formation^10^.

*In vivo*, AEP is a complex biological structure containing a significant proportion of naturally occurring peptides that originate from saliva^11^. Several studies have shown that specific salivary proteins such as histatin and statherin have a strong affinity for HA and prevent the precipitation of calcium and phosphate on the surface of the teeth, inhibiting unwanted biomineralization^4^. Thus, the identification of peptides in the AEP is of great interest because many salivary peptides exhibit functional domains that maintain the activity of the native protein. For example, among five naturally occurring AEP peptides derived from a small salivary protein statherin^11^, DR9, consisting of 9 amino acids DSpSpEEKFLR (where *Sp* is a phosphorylated serine), has shown a significant effect on HA crystal growth inhibition at all studied concentrations compared to other native statherin AEP peptides^12^. Another naturally occurring peptide found in the *in vivo* AEP derived from histatin 3 is RR14, which is a single functional domain represented by 14 amino acid residue domains located within the middle region of histatin 3, a critical antifungal protein^11,13^.

The functional domains DR9 and RR14 of the small proteins statherin and histatin 3, respectively, are not repeated in the primary structure of these proteins. Other salivary proteins have evolutionarily conserved one or more repeats of functional domains within their primary structure, thereby improving their functional capacity under evolutionary challenges^14^. Consequently, statherin and histatin 3, which are present in salivary secretions of humans and old-world monkeys, could be considered evolutionarily young, and artificially duplicating this peptide sequence (DR9) may induce the effects normally expected in the course of evolution.

Furthermore, other evolutionary processes have been known to occur resulting in the merging of functional domains from different proteins for a combinatory effect. While functional domain repeats augment a single functional characteristic, the combination of two distinct functional entities can lead to the development of constructs with multiple functions. This hybrid approach is particularly promising for pellicle components because some AEP peptides show high affinity for HA, whereas others have different functions such as antimicrobial properties^15,16^. Examples of such synthetic bi-functional constructs in the protein field are cystatin-histatin^17^ and statherin-osteopontin chimeras^18^.

In this study, by synthetically combining the DR9 and RR14 domains, we investigate the salivary protein evolution pathway and hypothesize the creation of a novel protein with enhanced mineral homeostatic properties and antimicrobial activity. Moreover, by duplicating the statherin functional domain DR9, we plan to create a more powerful peptide that inhibits calcium and phosphate crystal growth in the oral cavity.

## Results

Isoelectric points for each peptide were calculated at pH 6.8, the physiological salivary pH. DR9, the naturally occurring peptide derived from statherin, showed a pI of 3.63; DR9-DR9 showed the lowest pI value of 3.44, whereas RR14 showed the highest pI of 11.00. The hybrid peptide, DR9-RR14, showed a pI of 7.16 (Table 1).

**Table 1:** Constructed peptides derived from small salivary proteins statherin and histatin 3 and their calculated pI in pH 6.8.

Amino Acid Sequence (name)	Number of residues	MW	pI
DSpSpEEKFLR (**DR9**)	9	1270.10	3.63
DSpSpEEKFLRDSpSpEEKFLR (**DR9-DR9**)	18	2522.38	3.44
RKFHEKHHSHRGYR (**RR14**)	14	1875.10	11.00
DSpSpEEKFLRRKFHEKHHSHRGYR (**DR9-RR14**)	23	3127.29	7.16
DSpSpEEKFLRRIGRFGYGYGPYQPVPEQPLYPQPYQPQYQQYTF (**Statherin**)	43	5380.00	4.41
DSHAKRHHGYKRKFHEKHHSHRGYRSNYLYDN (**Histatin 3**)	32	4061.62	9.99

MW, molecular weight; pI, isoelectric point; Sp is a phosphorylated serine.

Regarding the inhibitory effect of the peptides on HA crystal growth, Table 2 summarizes the inhibition of HA crystal formation by all tested peptides in the concentration range of 0.9–9 µM. DR9-DR9 demonstrated the most significant inhibitory effect among the peptides and proteins at different concentrations (Table 2; *p* < 0.05). DR9 and statherin revealed similar inhibitory effects for all tested concentrations (*p* > 0.05), but significantly different from those of RR14, DR9-RR14, and histatin 3 in concentrations ranging from 1.8 to 5.4 µM (*p* < 0.05). Only at concentrations of 7.2 and 9.0 µM, DR9-RR14 demonstrated an inhibitory effect similar to that of DR9 and statherin. As expected, RR14 and histatin 3 showed a negligible inhibitory effect at all tested concentrations.

**Table 2:** Effect of constructed peptides derived from small salivary proteins statherin and histatin 3 in different concentrations on hydroxyapatite (HA) crystal growth inhibition.

	0.9 µM	1.8 µM	2.7 µM	3.6 µM	5.4 µM	7.2 µM	9.0 µM
DR9	1.78 ± 0.16^b^	1.56 ± 0.21^b^	0.85 ± 0.05^b^	0.80 ± 0.07^b^	0.63 ± 0.06^b^	0.53 ± 0.08^b^	0.24 ± 0.03^b^
DR9-DR9	1.09 ± 0.07^a^	0.68 ± 0.07^a^	0.54 ± 0.04^a^	0.50 ± 0.07^a^	0.43 ± 0.05^a^	0.28 ± 0.06^a^	0.12 ± 0.01^a^
RR14	2.04 ± 0.11^b^	2.00 ± 0.03^c^	1.97 ± 0.07^d^	2.00 ± 0.05^d^	1.99 ± 0.06^d^	1.99 ± 0.06^c^	2.01 ± 0.07^c^
DR9-RR14	1.98 ± 0.01^b^	1.79 ± 0.07^c^	1.64 ± 0.09^c^	1.20 ± 0.02^c^	0.94 ± 0.09^c^	0.59 ± 0.17^b^	0.23 ± 0.05^b^
STATHERIN	1.67 ± 0.18^b^	1.45 ± 0.11^b^	0.72 ± 0.06^b^	0.69 ± 0.10^b^	0.54 ± 0.06^b^	0.52 ± 0.04^b^	0.34 ± 0.07^b^
HISTATIN 3	2.02 ± 0.08^b^	2.04 ± 0.06^c^	1.92 ± 0.11^d^	1.93 ± 0.20^d^	1.92 ± 0.15^d^	1.94 ± 0.31^c^	1.94 ± 0.24^c^

The absorbance values measured at 560 nm represents a proxy for the amount of calcium formed in the hydroxyapatite crystal. Different letter(s) in superscripts indicate statistical difference, and same-letter superscripts indicate no statistical difference within the same column (P < 0.05). Standard deviation of the mean, calculated from 3 independent experiments.

When the data were presented based on the concentration efficiency, IC_50_ for all tested peptides and proteins was calculated (Table 3). Histatin 3 and RR14 failed to attain the IC_50_ value. DR9-RR14 reached the IC_50_ value at 3.80 µM. As expected, DR9 and DR9-DR9 demonstrated a considerable inhibitory effect on crystal growth by reaching the IC_50_ value at 2.82 µM and 1.07 µM, respectively. Statherin, reached the IC_50_ value at 2.50 µM (Table 3).

**Table 3:** The IC_50_ value of DR9, DR9-DR9, DR-RR14, Statherin. *Note*. RR14 and Histatin 3 did not reach a IC_50_ based on the 7 concentrations tested.

	IC_50_ (µM)
DR9	2.82
DR9-DR9	1.07
RR14	Not reachable
DR9-RR14	3.80
STATHERIN	2.50
HISTATIN 3	Not reachable

**Figure 1:**
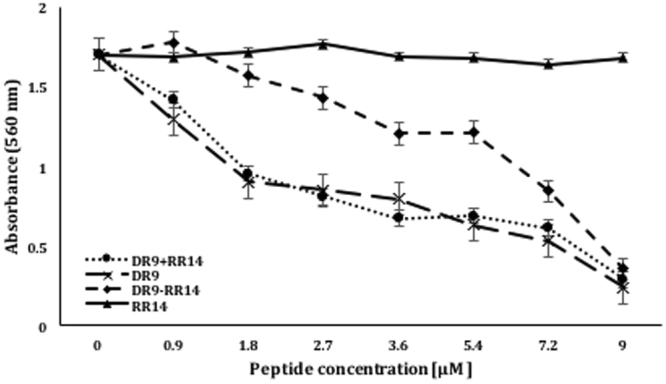
Plotting of the HA Inhibition effect when peptides DR9-RR14, DR9 and RR14 and a solution containing DR9 and RR14 (DR9 + RR14) were tested. Note. Bars represent standard deviation of the mean, calculated from 3 independent experiment.

The solution containing a mixture of DR9 and RR14 peptides showed results that were statistically similar to those obtained with DR9 at all seven tested concentrations. Notably, the values with the mixture of DR9 and RR14 were considerably more active than that of the hybrid peptide DR9-RR14 at concentrations from 0.9 to 7.2 µM. Only at the concentration of 9.0 µM, the activity of the DR9-RR14 peptide was statistically equivalent to that of the mixture of DR9 and RR14 peptides (Fig. 1, *p* > 0.05).

## Discussion

The importance of salivary peptides in oral homeostasis is becoming more evident owing to advanced research on naturally occurring peptides^19^. However, few studies have determined the functional roles of the newly identified salivary peptides and their potential biological effect upon modifications, with respect to a therapeutic point-of-view^15^. Peptides show significant pharmaceutical potential as an active therapeutic approach in several medical and biomedical areas such as endocrinology, urology, obstetrics, and oncology. Hence, it is necessary to design molecules from naturally occurring peptides that can manipulate disease-related biological targets for beneficial effects with low toxicity. However, naturally occurring peptides present limitations such as inadequate duration of action. In addition, an efficient therapeutic dose cannot be reached at physiological/pathological concentrations of the peptides. On the other hand, identification of these naturally occurring peptides opens avenues for the development of peptide-based drugs, where the protein evolutionary pathway is explored to eliminate challenges related to the stability of the harsh proteolytic environment and therapeutic dose.

Herein, we used naturally occurring AEP peptides to explore the protein evolutionary pathways and their effect on HA crystal growth inhibition. More specifically, we replicated the functional domain of DR9 and merged functional domains from two different proteins, statherin and histatin 3.

We chemically engineered the statherin domain (DR9), which has significant biological functions related to the inhibition of HA crystal growth that promotes enamel remineralization and inhibits dental calculus formation^12^. A duplication construct, DR9-DR9, was created. Moreover, a hybrid construct, DR9-RR14, was engineered from the biological domains of statherin and histatin 3. The results indicated that DR9-DR9 was significantly more active than the naturally occurring peptide, DR9, and its native protein (statherin). This result agrees with our prior study where DR9-DR9 was more efficient in prevent enamel demineralization than DR9 peptide^20^. The hybrid peptide, DR9-RR14 demonstrated moderate inhibitory ability when compared with other peptides and proteins. This observation is extremely relevant since the primary idea of the creation of a hybrid peptide is to merge biological functions of several peptide domains in a single peptide. In this case, we hypothesized that the effect of DR9 and/or statherin on the inhibition of HA crystal growth and that of RR14 and/or histatin 3 on antifungal activity could be merged in a single peptide, DR9-RR14. Antimicrobial actives of DR9-R14 was tested against S. mutans and C. albicans, demonstrating a significant effect on these microorganisms when compared with DR9, DR9-DR9^20^.

Based on the IC_50_ values, we observed that DR9-RR14 and DR9-DR9 required concentrations of at least 3.80 µM and 1.07 µM (almost four times less), respectively, to obtain the same effect. However, DR9-DR9 had a single biological function whereas DR9-RR14 showed the potential to retain multiple biological functions. DR9-DR9 demonstrated the strongest inhibitory ability, reaching IC_50_ at a concentration of 38% and 42% of that of statherin and DR9, respectively. We speculate that the observed phenomenon is based on the pI of these peptides measured at salivary pH and the degree of phosphorylation of these peptides. For example, the degree of phosphorylation of osteopontin affects its potential as an inhibitor^21,22^. Previous studies on statherin fragments^23^ showed that the mutation of both phosphoserines to a simple serine reduces the HA-binding affinity by almost 9-fold, indicating that the phosphoserine residues and acidic amino acid side chains are essential for the binding of statherin to HA. In relation to DR9, the presence of phosphoserine at positions 2 and 3 in DR9 resulted in a higher degree of HA inhibition than the other five unphosphorylated statherin peptides, including an identical peptide (DR9/2) lacking covalently linked phosphate at positions 2 and 3^12^. In this study, the peptide was not only phosphorylated at positions 2 and 3 but also at positions 11 and 12, and it showed a pI lower than that of the other tested peptides. These findings confirmed that phosphorylation plays a critical role in the mechanism of HA crystal growth in the oral cavity. Moreover, the presence of 4 phosphorylated sites in the DR9-DR9 suggests a more stable and strong binding conformation to the HA crystal. In prior study, we observed, by using molecular dynamic simulation, that the presence of phosphorylated sites in an identical amino acid chain can increase the binding affinity to HA crystals^12^. However, the molecular basis of this phenomenon requires further elucidation. Another aspect that needs attention in future studies is the ratio of number of molecules and molecular weight from peptides, such as DR9 and DR9-DR9, to produce similar HA growth inhibition. This can be observed with a simple comparison between DR9-DR9 with double concentrations of DR9 or statherin. When DR9-DR9 in a concentration of 1.8 uM is compared to DR9 and statherin in a concentration of 3.6 uM, it is very clear that DR9-DR9 still performs better than DR9, but the effects on the inhibition of crystal growth seem to be similar to that of statherin.

Oral clinical applications require the development of novel therapeutic approaches that can contribute to oral homeostasis. The most efficient methods to control dental caries and dental calculus remain mechanical in nature. These methods are primarily confined to tooth brushing and flossing techniques, which are only effective when carried out adequately on a daily basis. The mechanical means of plaque removal, however, are difficult to master consistently for a large segment of the population, such as in the growing section of the elderly as well as individuals with certain special needs. This study presents promising molecular methodologies for clinical exploitation in oral health maintenance. More specifically, the long-term vision is focused on generating peptides for clinical applications, since these can be chemically synthesized on a large scale and at reasonable production costs. It is important to mention that the primary focus of this study was not to generate a new drug but to investigate the biological ability of domain multiplication and hybridization, which was demonstrated successfully as observed in other research fields^24,25^. We recognize that the interplay between these constructed peptides and the oral environment is highly complex. Besides, it should be noted that additional tests such as toxicity testing using adequate animal models should be carried out before clinical trials. Overall, our data suggest that duplication of the functional domain could enable the creation of a salivary protein with enhanced capacity for maintaining dental homeostasis, such as high inhibition of calcium phosphate crystal formation in the saliva, which is an essential function for promoting dental remineralization and inhibition of dental calculus.

## Methods

### Characterization of the Engineered Novel Peptides

Engineered novel peptides derived from the functional domains of statherin and histatin 3—DR9, RR14, DR9-DR9, and DR9-RR14 were chemically synthesized by Chinapeptide (Shanghai, China), and all peptides are listed in Table 1. The purity (>95%) and molecular weight (MW) of the proteins and each peptide were verified by high-performance liquid chromatography analysis. Statherin and histatin 3 proteins were also chemically synthesized by Chinapeptide and included in this study as positive control and negative control proteins, respectively.

### Calculation of Peptide Isoelectric Points

The isoelectric points (pI) of the constructed peptides, statherin, and histatin 3 were determined using the calculator developed by Gauci *et al*.^26^. This approach calculates the pI of a protein and peptide at a particular pH by using user-specified pK values, and this calculation is repeated until the pH corresponding to a net charge of zero is attained. The pI values quoted were calculated using the Scansite option at pH 6.8.

### Microtiter Plate HA-inhibition Assay

A 96-well microtiter plate was coated with 80 µl peptides (of concentrations ranging from 0.9 to 9 µM) in a buffer containing 50 mM 4-(2-hydroxyethyl)-1-piperazineethanesulfonic acid (HEPES) and 150 mM NaCl (pH 7.4) at room temperature, 23 °C (RT) for 1 h. Thereafter, 10 µL of a solution containing 10 mM KH_2_PO_4_, 20 mM Na_2_HPO_4_, and 150 mM NaCl (pH 7.4) and 10 µL of a second solution containing 50 mM CaCl_2_, 50 mM HEPES, and 150 mM NaCl (pH 7.4) were added to each well. The resulting 100-µL solution was incubated at RT for 4 h for HA crystal formation. After this period, the solution was carefully removed using a micropipette, keeping the HA crystals at the bottom of the well. Furthermore, 5% alizarin red S (pH 4.2) was added to the well and incubated for 1 min to stain the calcium ions present in the HA crystals. Immediately after, the stain solution was removed using a micropipette, and 100 µL of 100 mM cetylpyridinium chloride was added to each well and incubated at RT for 1 h. A control sample well was treated in the same way as above, without the addition of protein or peptides. Calcium ions from the HA crystals formed during the experiment were analyzed spectrophotometrically at 560 nm by using a microplate reader to evaluate the effect of the tested peptides on HA inhibition^12^. In addition, IC_50_ value was calculated for all tested constructed peptides.

An additional experiment was performed to verify the potential synergistic effect of HA crystal growth inhibition when DR9 and RR14 share the same environment. A buffer solution containing 50 mM HEPES and 150 mM NaCl (pH 7.4) containing an equal number of DR9 and RR14 molecules was incubated at RT for 1 h. Final concentrations ranging from 0.9 to 9 µM of DR9 and RR14, DR9, RR14, and DR9-RR14 were used here. After this initial peptide incubation, experiments for HA crystal growth inhibition were performed as described above^12^.

### Statistical analysis

ANOVA and a Student-Newman-Keuls test for pair-wise comparisons performed to compare the peptide groups. A *p* value of <0.05 was considered statistically significant.
